# Microsatellite Instability: A Review of Molecular Epidemiology and Implications for Immune Checkpoint Inhibitor Therapy

**DOI:** 10.3390/cancers15082288

**Published:** 2023-04-13

**Authors:** Alexandra Kavun, Egor Veselovsky, Alexandra Lebedeva, Ekaterina Belova, Olesya Kuznetsova, Valentina Yakushina, Tatiana Grigoreva, Vladislav Mileyko, Mikhail Fedyanin, Maxim Ivanov

**Affiliations:** 1 OncoAtlas LLC, 119049 Moscow, Russia; 2 Department of Evolutionary Genetics of Development, Koltzov Institute of Developmental Biology of the Russian Academy of Sciences, 119334 Moscow, Russia; 3 Faculty of Physics, Lomonosov Moscow State University, 119991 Moscow, Russia; 4 N.N. Blokhin Russian Cancer Research Center, 115478 Moscow, Russia; 5 Laboratory of Epigenetics, Research Centre for Medical Genetics, 115522 Moscow, Russia; 6 Shemyakin-Ovchinnikov Institute of Bioorganic Chemistry of the Russian Academy of Sciences, 117997 Moscow, Russia; 7 State Budgetary Institution of Health Care of the City of Moscow “Moscow Multidisciplinary Clinical Center” “Kommunarka” of the Department of Health of the City of Moscow, 142770 Moscow, Russia; 8 Federal State Budgetary Institution “National Medical and Surgical Center named after N.I. Pirogov” of the Ministry of Health of the Russian Federation, 105203 Moscow, Russia; 9 Moscow Institute of Physics and Technology, 141701 Dolgoprudny, Russia

**Keywords:** microsatellite instability, mismatch repair, biomarker, precision medicine, immune checkpoint inhibitors

## Abstract

**Simple Summary:**

Microsatellite instability (MSI) occurs in a wide variety of tumor types and is one of the most important predictive biomarkers for immune checkpoint inhibitor therapy. The present review aims to summarize the molecular characteristics of MSI tumors as well as the results of major clinical studies evaluating the efficacy of checkpoint inhibitors in the treatment of patients with MSI tumors.

**Abstract:**

Microsatellite instability (MSI) is one of the most important molecular characteristics of a tumor, which occurs among various tumor types. In this review article, we examine the molecular characteristics of MSI tumors, both sporadic and Lynch-associated. We also overview the risks of developing hereditary forms of cancer and potential mechanisms of tumor development in patients with Lynch syndrome. Additionally, we summarize the results of major clinical studies on the efficacy of immune checkpoint inhibitors for MSI tumors and discuss the predictive role of MSI in the context of chemotherapy and checkpoint inhibitors. Finally, we briefly discuss some of the underlying mechanisms causing therapy resistance in patients treated with immune checkpoint inhibitors.

## 1. Introduction

Microsatellite instability (MSI) results from impaired DNA mismatch repair (MMR) and causes an accumulation of mutations in microsatellites (MS), also called short tandem repeats (STRs). STRs consist of repeated sequences of 1–6 nucleotides and account for 3% of the genome, both coding and noncoding regions [1,2,3]. The nature of MS determines its outstanding tendency to accumulate errors. It happens due to DNA slippage in the process of DNA replication, which usually leads to a change in MS length [4,5].

The MMR system is highly conserved across species. MMR is responsible for the recognition and correction of mismatched nucleotides and plays a key role in maintaining genomic stability [6,7,8]. Four major proteins encoded by the *MLH1* (mutL homologue 1) [9,10], *MSH2* (mutS homologue 2) [11], *MSH6* (mutS homologue 6), and *PMS2* (postmeiotic segregation increased 2) [12] genes play a central role in this process [13]. The MMR system functions through the formation of heterodimers (hMutS and hMutL). hMutS consists of MSH2 and one of the secondary proteins, either MSH6 or MSH3, and recognizes mismatched nucleotides and small indels [7,8]. The hMutL heterodimers, consisting of MLH1 and one of the secondary proteins, PMS2, PMS1, or MLH3, participate in MMR reactions [14,15,16] via its endonuclease activity [17], 5′ nicking [18], modulation, and termination of exonuclease 1′s (Exo1) activity [18,19,20]. Thus, hMutL deficiency leads to Exo1 hyperactivity and increased DNA excision [19,20]. The inactivation of at least one of the following genes: *MLH1*, *MSH2*, *MSH6*, or *PMS2*, due to germline and/or somatic mutations or epigenetic silencing, results in the MMR system deficiency (dMMR) [21,22].

MSI occurs among various tumor types, and is most common in colorectal, small bowel, endometrial, and gastric cancers [3,23,24,25,26,27,28,29]. Most cases of MSI are sporadic, arising from epigenetic inactivation of MLH1 gene expression. However, the MSI can also be caused by Lynch syndrome, a hereditary condition resulting from germline pathogenic mutations in the MMR genes, coupled with inactivation of the second allele [30,31,32].

MSI is the one of the major biomarkers predictive of the immune checkpoint inhibitor (ICI) benefit across cancer types, both in Lynch syndrome-related and sporadic tumors. ICI therapy aims to overcome tumor immune escape through targeting immune inhibitory molecules (e.g., PD-1, PD-L1, LAG3, and CTLA4) expressed on the surfaces of tumor and immune cells [33,34,35]. Correct assessment of MSI is critical for adequate therapeutic decisions. According to ESMO recommendations [30], several methods are used in clinical practice to assess MSI status. The IHC method indirectly assesses MSI by detecting loss of staining for MLH1, MSH2, PMS2, and MSH6 proteins. An advantage of using IHC to detect MMR proteins is its convenience and ability to identify the target gene for future mutational confirmation [24]. PCR-based approaches directly determine MSI status via amplification of specific microsatellite repeats. ESMO suggests PCR in case of indeterminate IHC results, including disagreement or difficulties in interpreting IHC. The five poly-A mononucleotide repeats panel (BAT-25, BAT-26, NR-21, NR-24, NR-27) is considered the current standard [30], though the Bethesda panel, comprising of two mononucleotide (BAT-25 and BAT-26) and three dinucleotide (D5S346, D2S123, and D17S250) repeats, is also widely utilized in clinical practice [36,37]. In diagnostics, MSI (previously known as MSI-H) denotes alterations in the lengths of several MS (e.g., 2 of 5 loci in a standard PCR test with five poly-A mononucleotide repeats). In contrast, if the number of unstable loci does not exceed one, it is termed as MSS (microsatellite stable). NGS-based MSI detection is considered as one of the most promising, since its advantages include higher accuracy and an expanded spectrum of microsatellites analyzed, which is relevant for non-CRC tumors that can harbor a non-standard set of unstable microsatellites [38]. NGS also allows simultaneous analysis of a comprehensive spectrum of clinically significant biomarkers [14,30].

The first FDA-approved ICI drug was ipilimumab, which was initially approved in 2011 for the treatment of melanoma and is now used in a limited range of cancers. Pembrolizumab is a second FDA-approved ICI, which has the widest range of indications, including tissue-agnostic indications [39]. To date, more than 6 ICI have been approved for site-specific or tumor-agnostic indications. Overall, the objective response rate (ORR) for tumors with MSI varies between 34% and 69% depending on the line of therapy, while the rate of pathologic complete response (pCR) may reach 100% in neoadjuvant settings, which indicates a high efficacy of checkpoint inhibitors in MSI tumors [40,41,42]. At the same time, about a quarter of metastatic colorectal cancers and up to half of some other types of cancer are intrinsically resistant to ICI [43,44]. Several mechanisms of such an intrinsic resistance have been proposed, including the activating mutations in the RAS/RAF signaling pathway [45], mutations in the antigen presentation machinery and interferon pathway genes [46,47], establishment of an immunosuppressive microenvironment [48], as well as the influence of the gut microbiome [49] and immunoediting theory [50].

Here, we describe the prevalence of MSI among various types of cancer, as well as summarize the data on the application of MSI as a biomarker for the efficacy of ICI. Specifically, the clinical studies of patients with colorectal cancer, as well as patients with endometrial and gastric cancer, are reviewed. We also discuss potential mechanisms of resistance to ICI, limiting the response to therapy in tumors with MSI/dMMR.

## 2. Lynch Syndrome-Associated Cancer Risks

Lynch syndrome is an autosomal-dominant inherited cancer syndrome caused by pathogenic or likely pathogenic germline mutations in genes of the MMR system [9,11,51,52,53,54,55,56]. A large cohort study estimated that a pathogenic variant in one of the MMR genes occurs in 1 out of 279 people in the general population [57]. In Lynch syndrome, MSI is mainly driven by germline mutations in *MSH2* (up to 40–45% in colorectal and endometrial cancers). The second place takes the mutations in *MLH1* (up to 40% in colorectal and 30% in endometrial cancer) and *MSH6* (up to 5–10% in colorectal and 30% in endometrial cancer). *PMS2* mutations are the rarest event in Lynch syndrome-associated MSI (up to ∼5–10% in colorectal and mostly absent in endometrial cancer) [58,59,60]. Additionally, up to 3% of Lynch syndrome-associated MSI cases are caused by rare germline 3′ end deletions of the last exons of the epithelial cell adhesion molecule (*EPCAM*) gene that lead to epigenetic hypermethylation of the *MSH2* promoter located downstream of *EPCAM* [54,61,62].

The presence of pathogenic variants in any of the MMR genes (*MLH1*, *MSH2*, *MSH6*, *PMS2*, or *EPCAM*) is associated with an increased risk of developing various cancer types. Among these Lynch-associated cancer types, colorectal, endometrial, ovarian, gastric, small bowel, and pancreatic cancers are most frequently described [36,63,64,65,66,67,68]. Rarer types of cancer that are at slightly increased risk among individuals with Lynch syndrome include renal pelvis and/or ureter, bladder, biliary tract, prostate, breast, central nervous system, and even skin cancer [64,69,70,71,72,73,74].

Carriers of pathogenic variants of the MMR genes are most susceptible to the development of colorectal cancer; historically, Lynch syndrome is also known as hereditary nonpolyposis colorectal cancer syndrome. According to a meta-analysis by Wang et al. [75], which assessed the risk of developing colorectal cancer by age 70, for carriers of pathogenic variants of *MLH1*, *MSH2*, and *MSH6*, the risk was 44%, 54%, and 12%, respectively, for men, and 37%, 39%, and 12%, respectively, for women. For carriers of pathogenic *PMS2* variants, this risk is about 8–20% [76,77,78]. There is significant heterogeneity of the reported risks in the literature [63,64,65], as well as in the NCCN guidelines [79]. Overall, the risk of developing colorectal cancer for carriers of pathogenic MMR gene variants is several times greater than the cumulative lifetime risks for the general population, which accounts to 4% [80]. For women affected by Lynch syndrome, the risk of developing endometrial cancer is also elevated [63,66]. According to various studies, for carriers of pathogenic variants *MLH1*, *MSH2*, *MSH6,* and *PMS2*, the risk of developing uterine cancer is 34–54%, 21–57%, 16–49%, and 13–20%, respectively [63,64,66,76,77,81,82], which is also significantly higher than the population average risk of 1.3% [80].

The risks of developing other common Lynch-associated cancers, such as ovarian, gastric, and small bowel cancers, are generally significantly higher for carriers of pathogenic *MLH1* and *MSH2* variants compared to those for individuals harboring pathogenic mutations in the *MSH6* and *PMS2* genes. Thus, according to various estimates, the risk of developing ovarian cancer reaches 4–20% for *MLH1* carriers and 8–38% for *MSH2* but is only 1–13% for *MSH6* and up to 3% for *PMS2* [63,64,67,81]. A similar trend is observed for the risks of developing gastric and small bowel cancers. For example, for carriers of MLH1 pathogenic variants, there is a ten-fold increase in the risk of developing gastric and small bowel cancer, although this risk is not as high as for other previously mentioned cancer types, 5–7% and 0.4–11%, respectively [63,64,67,68]. For all other types of cancer, the estimation of risks of developing cancer is either insignificant or inconsistent between studies, resulting in a wide range of values obtained, often not exceeding the risks for the general population.

**Figure 1:**
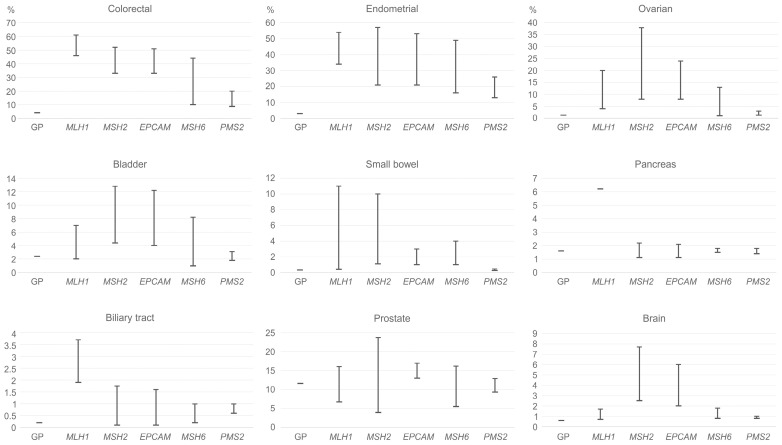
Lynch syndrome-associated cancer risks by age 80, according to the NCCN guidelines. GP = general population.

Gene-specific Lynch syndrome cancer risks based on data from the NCCN guidelines [79] are shown in Figure 1.

## 3. MSI: Molecular Epidemiology across Cancer Types

**Figure 2:**
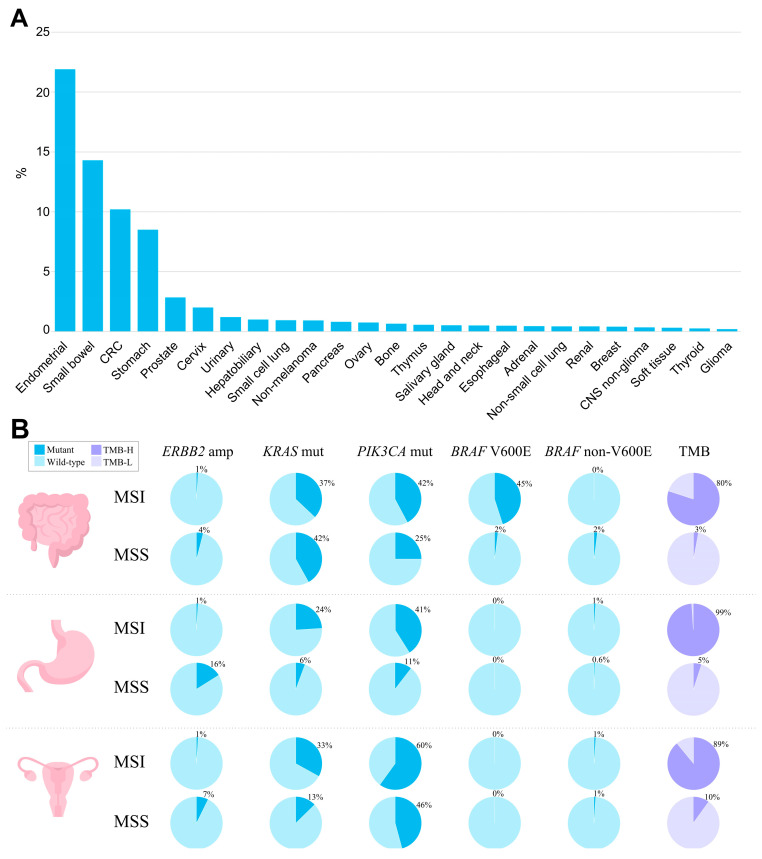
The landscape of MSI and genomic characteristics of MSI tumors across cancer types, according to TCGA. (**A**) Prevalence of MSI (%) across tumor types. (**B**) Prevalence of genomic alterations typically found in MSI and MSS tumors in colorectal adenocarcinoma, stomach adenocarcinoma, and uterine corpus endometrial carcinoma.

The MSI phenotype can be found in many cancer types. In a study analyzing more than 11,000 tissue samples from patients with 39 cancer types, MSI was found in 27 tumor types (overall in 3.8% of all samples) [83]. The tumor types where the MSI phenotype is observed include colon, gastric, endometrial, ovarian, hepatobiliary tract, urinary tract, brain, and skin cancers (Figure 2A). Of those, the highest prevalence of MSI is in colorectal cancer (10.2%, range 6.6–14.5%) [23,24,30,84,85,86,87,88,89,90,91], endometrial cancer (especially endometrioid histotype) (21.9%, range 15.1–29.6%) [25,26,27,84,92,93,94], gastric cancer (8.5%, range 6.4–10.9%) [3,27,28,30,84,95], and small bowel cancer (14.3%, range 5.4–26.3%) [84]. In gastric cancer, the frequency of MSI/dMMR varies significantly within histological subtypes: from 0.9% in the mixed-type and 2.9% in the diffuse-type, to 10.7% of the intestinal-type [28,96]. In other types of cancer, the MSI rate is relatively low [84], specifically 2–10% in ovarian [30,60,97,98] and only 1–2% in pancreatic cancer [30,99,100,101]. The assessments of the MSI rate in urothelial carcinoma are highly contradictory, with the reported values ranging from 1% to as high as 46% [102,103,104]. The MSI phenotype is also reported in Lynch syndrome-unrelated cancer types—in glioblastoma, cervical cancer, small intestine, melanoma, sarcoma, and others, but in these cancer types, MSI is much rarer [30,105,106].

MSI is characteristic of both Lynch syndrome-associated cancer, where it is observed in nearly all cases, and sporadic cancer, where it reaches up to 10–15% of cases [91]. Conversely, Lynch syndrome comprises not more than 19% of MSI cancers, with the highest rate in MSI-positive colorectal cancer, followed by endometrial (5–10%), small bowel (12%), and gastric (4–15%) [96,106,107,108,109,110,111,112]. Respectively, most MSI cases (80% to 95%) arise sporadically [113]. The major cause of MSI is promoter hypermethylation of both MLH1 gene alleles, leading to a loss of MLH1, which is observed in ~90% of sporadic cancer [23,36,58,113,114,115].

When studying the molecular profiles of MSI tumors regardless of whether the tumor is Lynch syndrome-associated or sporadic, a number of oncogenic mutations can be found, including mutations in *KRAS*, *NRAS*, *BRAF*, *PIK3CA*, *APC*, *TP53*, etc. Specifically, *KRAS* is observed in MSI tumors with a frequency of 30–37% in endometrial, small bowel, and colorectal cancers, and at 15–28% in gastric cancer [29,86,96,116,117,118]. In colorectal cancer, the prevalence of *KRAS* mutations in MSI cases is lower than in MSS, where it reaches 46% [119]. Notably, MSS tumors harboring *KRAS* oncogenic mutations are characterized by more aggressive growth [115,120].

*BRAF* mutations are found with a high frequency (up to 45%) in MSI colorectal cancer, with mostly exclusive prevalence of p.V600E [121,122]. *BRAF* mutations have strong bias to sporadic cases, and mostly can never be observed in hereditary colorectal cancer [122,123,124,125]. According to Parsons et al.’s meta-analysis [126], *BRAF* V600E variants occur in only 1.4% of patients with Lynch syndrome. In sporadic colorectal carcinomas displaying the MSI phenotype, *MLH1* hypermethylation and *BRAF* p.V600E mutations frequently co-occur, indicating a possible causal relationship between *BRAF* mutations and MLH1 loss [127,128,129]. However, the straight relationship between *MLH1* hypermethylation and *BRAF* p.V600E mutation might be called into question by the fact that not all colorectal cancers with *BRAF* p.V600E mutations display silenced *MLH1* with subsequent MSI. Such tumors remain microsatellite-stable, suggesting that other factors can influence the MSI phenotype in *BRAF*-positive cancer [130]. Indeed, only 20–30% of *BRAF* p.V600E-mutated metastatic colorectal cancer display MSI [131]. Additionally, the transition to the MSI phenotype via *MLH1* hypermethylation is observed in approximately 75% of *BRAF*-mutated sessile serrated adenomas, with the remaining cases developing into MSS cancers. Among traditional serrated adenomas, *BRAF* mutations occur in two-thirds of the cases, but *MLH1* silencing and the MSI phenotype are rare [132,133]. In other cancer types, the interplay between *BRAF* p.V600E and MSI/dMMR is not observed [3,27,94,134,135].

*TP53* mutations are much less frequently observed in MSI as compared to MSS tumors, and their frequency in colorectal and gastric MSI cancer is 20–30%, as compared to 50–65% among MSS tumors [3,136]. Similarly, in the endometrial, pancreatic, and ovarian cancers, where TP53 mutations are common events, MSI tumors harboring *TP53* can rarely be found [94,99,100,137]. This pattern of *TP53* distribution among MSI and MSS tumors may indicate that *TP53* mutations are unlikely to contribute to the MSI cancer tumorigenesis.

The prevalence of *PIK3CA* mutations in colorectal and gastric tumors is higher in MSI tumors (30–45%), as compared to MSS (10–25%). However, in endometrial cancer, *PIK3CA* mutations are equally common among both MSI and MSS tumors, accounting for 45–60% of all cases [94].

Other frequently altered genes in MSI tumors include *RNF43*, *ATM*, *ARID1A*, *BRCA2,* and *PTEN*. Mutations in these genes have been shown to have a several-fold increased mutation frequency in MSI colorectal carcinomas compared to MSS [138]. Approximately one in five cases of MSI tumors have important targetable fusions in *NTRK1/2/3*, *ALK*, or *RET* genes [138], but there is no significant relationship between MSI and *HER2* amplification [139,140].

In the co-occurrence of MSI and other oncogenic alterations, the role of dMMR and its onset in Lynch syndrome colorectal carcinomas is described with three models [4]. In the first (classical) model, MMR deficiency is a secondary event that occurs after adenoma development, initially driven by somatic oncogenic mutations in *APC* and *KRAS* [141,142]. Distinguishing features of adenomas developing in accordance with this model are MMR proficiency and MSS. The classical model is typically observed in patients carrying germline *MSH6* or *PMS2* mutations [143]. It is known that in case of isolated loss of MSH6, MMR activity can be retained due to overlapping functions with MSH3, which explains the relatively low risk of cancer for *MSH6* mutation carriers [144]. Notably, *MLH1* and *MSH2* mutation carriers rarely develop tumors via the classical model [141]. The prevalence of adenomas developing via this model of carcinogenesis can be roughly estimated at 25% [145,146,147]. In the second and third models, biallelic inactivation of MMR genes leading to dMMR is a driver event, and therefore MSI is observed in all such tumors [141,145]. The second model is mainly observed in *MLH1* and *MSH2* mutation carriers and is typically characterized by inactivation of tumor suppressors involved in the WNT pathway, predominantly *TGFBR2* and *RNF43*, due to frameshift mutations within microsatellites in the corresponding genes [138,147]. The third model accounts for about 10% of LS-associated colorectal carcinomas and is exclusively observed in patients with MLH1 mutations. Here, dMMR is accomplished with mutations in *CTNNB1* and *TP53* [141].

The spectrum of potentially actionable alterations typically observed in MSI and MSS tumors based upon data generated by the TCGA Research Network [148] is summarized in Figure 2B.

Another important aspect worth mentioning is the relationship between MSI and tumor mutation burden (TMB). dMMR leads to a hypermutator phenotype and increased TMB, which is believed to make tumors more immunogenic. Considering all solid tumors, the simultaneous presence of MSI and TMB is quite rare and occurs only in about 3–7% of cases [30,149]. However, in tumors associated with Lynch syndrome, the overlap between TMB and MSI becomes more significant. The rate of TMB-H (≥10 mutations/megabase) patients among MSI cases is estimated at around 80–100% in colorectal cancer [84,150,151], 83–93% in endometrial cancer [84,152,153,154], and almost 100% in gastric and small bowel cancer [29,84,155]. In colorectal carcinoma, tumors with simultaneous presence of a high TMB and MSI/dMMR correspond to the CMS1 subtype [156], which is characterized by hypermutation, hypermethylation, enrichment in *BRAF* V600E mutations, as well as a strong infiltration of the tumor microenvironment with immune cells [88,157,158]. In this subtype, hypermethylation of the promoter regions of the *MLH1* gene leads to its silencing, the accumulation of DNA mutations, and the expression of neoantigens that contribute to the high immunogenicity of the tumor [159]. The TMB levels in MSI tumors are likely dependent on certain MMR complex loss and tumor histology/primary site [160,161]. According to a study by Salem et al., evaluating colorectal, endometrial, and other tumors, overall, the loss of mutSα (MSH2/MSH6) leads to a more pronounced TMB than the loss of mutLα (MLH1/PMS2). However, in some types of tumor histology, secondary DNA repair pathways can better mitigate dMMR, resulting in a less pronounced TMB under the same IHC protein loss patterns. These findings support the diversity of gene- and histologically-specific heterogeneity of MSI/dMMR tumors [149]. Patients with MSI displaying a high TMB have a better prognosis and are also good candidates for checkpoint inhibitor therapy [159].

Three predictive biomarkers are currently used to select subgroups of patients eligible for ICI immunotherapy: PD-L1 expression, MSI/MMR status, and TMB.

Initially, it was considered that a common mechanism of tumor immune evasion is the aberrant expression of immune inhibitory molecules, PD-L1, on the surface of cancer cells [162]. However, over time, evidence has emerged that even in the absence of PD-L1 expression, tumors often remain sensitive to ICI [162,163]. Additionally, TMB-H and MSI tumors respond to the immunotherapy regardless of PD-L1 expression [164]. To some extent, it can be explained by the focal expression of PD-L1 [165], which can be missed during needle biopsy, or by the dynamic and inducible nature of PD-L1 expression [166]. This led to the PD-L1 expression-independent indications of ICI for many types of cancers. While anti-PD-L1 therapy acts to overcome local immune resistance, CTLA-4 is expressed in T-cells and acts non-locally, often causing autoimmune reactions and lymphocyte invasion in different unaffected organs [167,168]. For that reason, prescription of anti-CTLA4 therapy does not require determination of the CTLA4 expression status.

dMMR is the cause of the hypermutator phenotype and increased TMB, which likely leads to increased tumor immunogenicity. Indeed, the connection between MMR phenotype, TMB, and tumor immunogenicity is more sophisticated since some methods of TMB calculation may exclude truncating mutations in certain genes [169], while the dMMR phenotype is characterized by an increased frequency of frameshift mutations producing an abundance of highly immunogenic neoantigens [170,171,172]. Thus, dMMR/MSI status should be considered as an independent predictive biomarker of ICI effectiveness, and the presence of a TMB-H in this subgroup may be an additional factor indicating an increased immunogenicity of the tumor.

## 4. MSI and Chemotherapy

For many years, chemotherapy was the only treatment option for patients with advanced colorectal cancer. It has been well-established that MSI/dMMR status is a good prognostic factor for patients with early-stage (II–III) colorectal cancer [173,174,175,176]. Although, it was subsequently confirmed that while MSI status seems to be a good prognostic factor in low-risk stage III patients (T1-3, N1), it is no longer so in high-risk stage III patients (T4 and/or N2) [177,178]. For patients with stage IV metastatic colorectal cancer, the prognostic impact of MSI status, on the contrary, becomes worse than for those with an MSS tumor [121].

However, as for the role of MSI on chemotherapy response, the predictive role is ambiguous. Several studies have reported data supporting MSI as a predictive biomarker for improved response to irinotecan-based chemotherapy [179,180]. Conversely, an in vitro study found that patients would need an intact MMR system to induce apoptosis of fluorouracil-modified DNA [181]. Consistent with the results of the in vitro study [181], a negative predictive effect of MSI/dMMR status in relation to 5-fluorouracil adjuvant chemotherapy was also reported in a retrospective study [182]. Additional data indicating a negative effect of adjuvant chemotherapy used to treat patients with colorectal cancer has been described in the literature [183]. However, most studies, predominantly retrospective, have found no predictive effect of MSI/dMMR in response to fluorouracil- or oxaliplatin- or irinotecan-based therapy (predominantly FOLFOX or FOLFIRI combinations) [173,174,184,185,186,187,188,189,190]. Innocenti et al. reported results for patients with metastatic colorectal cancer (mCRC), showing no difference between microsatellite instability status in patients treated with bevacizumab as a first-line therapy, but a shorter OS and PFS in MSI mCRC patients treated with cetuximab as compared to MSS mCRC patients. Better outcomes rates were also demonstrated in the MSI subgroup of patients treated with bevacizumab compared to those treated with cetuximab [191].

Thus, data on the predictive role of MSI in relation to chemotherapy efficacy are inconsistent. Fortunately, ICIs have become the standard of care for tumors exhibiting features of MSI, which has reduced the relevance of this issue for chemotherapy.

## 5. MSI as a Predictive Biomarker for ICI Efficacy

Cancer immunotherapy in the broad sense is the type of cancer treatment aimed to overcome one of the hallmarks of cancer—avoiding immune destruction. One of the mechanisms utilized by cancer cells for this purpose is the expression of immune inhibitory molecules on their surfaces or the recruitment of immune cells expressing these molecules. This class of molecules is called immune checkpoints, and in normal conditions serves to avert autoimmunity. They can be targeted with monoclonal antibodies known as ICIs. The anti-CTLA4 drug ipilimumab became the first ICI approved by the FDA in 2011. The second FDA-approved ICI is an anti-PD-1 drug, pembrolizumab, which has the widest range of indications within ICIs, including tissue-agnostic indication tumors with signs of increased immunogenicity. In current clinical practice, four different immune checkpoints can be targeted by several ICIs: PD-1 by four drugs, PD-L1 by three drugs, LAG3 by relatlimab, and CTLA4 by ipilimumab and tremelimumab (Table 1). CTLA4 inhibitors are almost always used in combination with anti-PD-(L)1 ICIs, while relatlimab is currently used only in combination with nivolumab.

**Figure 3:**
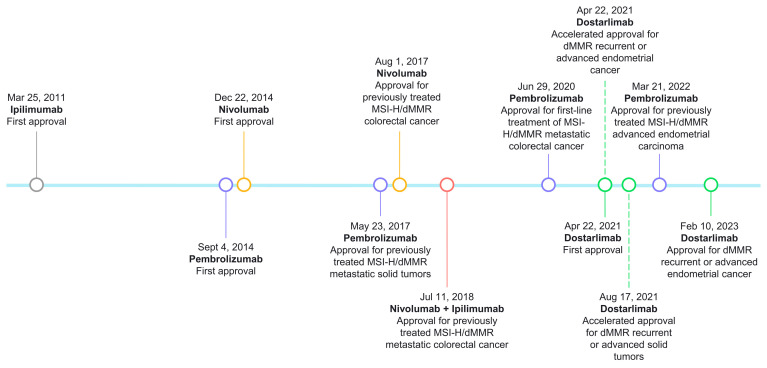
FDA approval timeline of immune checkpoint inhibitors for MSI/dMMR tumors.

The milestones of ICIs’ approval for MSI/dMMR tumors are presented in Figure 3.

### 5.1. Site-Agnostic Indications and Clinical Evidence

On 23 May 2017, the anti-PD-1 drug pembrolizumab became the first cancer drug approved by the FDA for tissue/site-agnostic use in patients with unresectable or metastatic MSI or dMMR solid tumors. Approval was based on the results of five different clinical trials: KEYNOTE-016, KEYNOTE-164, KEYNOTE-012, KEYNOTE-028, and KEYNOTE-158. In total, 149 patients with dMMR/MSI were recruited, 89 of them had colorectal cancer (CRC), 14 had endometrial cancer (EC), and 45 had other types of cancers. ORR across tumor types was 39.6%, with a complete response rate of 7.4% and a partial response rate of 32.2% [39]. More recent results from the KEYNOTE-158 study involving 233 patients with MSI/dMMR non-colorectal cancer showed a similar ORR of 34.3%, with median progression-free survival (PFS) of 4.1 months and median overall survival (OS) of 23.5 months [40].

In 2021, another anti-PD-1 drug—dostarlimab—received FDA accelerated approval for tumor-agnostic use in dMMR tumors based on the results of the GARNET trial. According to the drug label, 209 patients took part in the study, 103 of them had EC, 69 had CRC, and 37 had other types of cancers. The ORR was 38.7% in non-endometrial cancer patients [192]. The most recently published interim analysis showed that across 204 patients with non-endometrial cancer and dMMR/MSI or *POLE* mutations, the ORR reached 43.1%, and the probability of PFS at 6, 9, and 12 months was 51.8%, 48.1%, and 46.4%, respectively [193].

It should be noted that these studies have certain limitations, as they are non-randomized and uncontrolled, and majority of cancer types are represented by few or single patients. However, the high rate of responses observed in patients with exhausted standard therapeutic options allowed for the approval of these indications. Since these studies were performed in heavily pretreated patients, it can be expected that the use of ICI in earlier-line settings will be more effective. This assumption has been confirmed for colorectal cancer, which will be discussed below.

### 5.2. Site-Specific Indications and Clinical Evidence

#### 5.2.1. Colorectal Cancer

The most abundant data for site-specific use in dMMR/MSI tumors have been collected for CRC.

1. Pembrolizumab

The first observation of a MSI CRC patient having a three-year complete response to pembrolizumab therapy was published in 2013 [194]. More data on pembrolizumab efficacy in MSI CRC were collected in the KEYNOTE-016 trial. There were no objective responses in the cohort of 18 patients with MMR-proficient tumors [195]. Final analysis revealed that 21 of 40 (52%) MSI CRC patients responded to the therapy and 12% of patients achieved a complete response. No statistically significant difference in major endpoints was observed among patients with or without Lynch syndrome-associated tumors: ORR was 46% vs. 59% (*p* = 0.27), respectively, and hazard ratios for PFS and OS were 1.2 (*p* = 0.61) and 1.71 (*p* = 0.24), respectively. All patients were pretreated and had a progressive disease before entering the trial [105].

Results of the phase III randomized KEYNOTE-177 trial evaluating pembrolizumab efficacy in the first-line settings showed an ORR of 45% (*n* = 69) in patients receiving pembrolizumab and an ORR of 33% (*n* = 51) in patients receiving chemotherapy. Complete response rates were 13% and 4%, respectively. Median PFS was 16.5 months in pembrolizumab and 8.2 months in chemotherapy groups (HR 0.59). No statistically significant differences in OS were demonstrated, presumably due to the high crossover frequency between chemotherapy and pembrolizumab groups. Subgroup analysis did not reach sufficient statistical power to show the difference between *BRAF* or *RAS* mutant and wild-type tumors [196]. However, in an earlier publication of the results of this study, *RAS* mutant patients were shown to derive no benefit in terms of PFS. There was also a higher incidence of progressive disease in the pembrolizumab group compared to the control group (29% vs. 12%) [197]. Based on the interim results of this trial, pembrolizumab was approved by the FDA for the first-line treatment of patients with MSI CRC in 2020 [198].

A recent study of pembrolizumab in a neoadjuvant setting in 35 patients with localized or locally advanced solid tumors with MSI included 27 CRC patients. Here, 33 patients eligible for radiographic response evaluation had an ORR of 82%. Among 14 CRC patients who underwent surgery, 11 had a pCR (79%). Subgroup analysis did not reveal a difference in treatment efficacy between sporadic and Lynch-associated MSI [199].

2. Nivolumab and Ipilimumab

Nivolumab with or without ipilimumab is another FDA-approved therapeutic option for dMMR/MSI CRC, with the approval being based on the positive results of the CheckMate-142 uncontrolled trial [200,201]. Long-term survival analysis showed that nivolumab monotherapy results in an ORR of 34% and disease control rate (DCR) of 62% in pretreated patients (*n* = 74), with a median PFS of 6.6 months and 1-year survival of 72%. Patients (*n* = 21) who received 2 or less standard chemotherapy regimens achieved an ORR of 52% and DCR of 81% [202], indicating greater efficacy in earlier lines of therapy. Follow-up from CheckMate-142, a study evaluating nivolumab plus low-dose ipilimumab in patients who had more than one previous line of therapy, showed an ORR of 65%. The 24-month PFS and OS rates were 63% and 75%, respectively [203]. The most recent results from CheckMate-142 were obtained on the cohort treated in the first line with nivolumab plus low-dose ipilimumab. The ORR was 69%, with a complete response rate of 13%. The 24-month PFS and OS rates were 74% and 79%, respectively. *BRAF* and *KRAS* mutation status did not affect the clinical benefit [41].

The GERCOR NIPICOL study aimed to evaluate the optimal duration of immunotherapy for patients with MSI/dMMR CRC previously treated with fluoropyrimidine, oxaliplatin, and irinotecan, with or without targeted therapies. The patients were administered nivolumab plus ipilimumab for one year. Three-year PFS and OS rates were 70% and 78%, respectively [204].

Nivolumab in combination with ipilimumab was tested in neoadjuvant settings in patients with MMR-deficient and -proficient early-stage colon cancers in the exploratory NICHE study. All 20 patients with dMMR tumors achieved a pathological response, of which 19 had a major pathological response (≤10% residual viable tumor) and 12 had a complete pathological response. Only 4 of 15 patients with MMR-proficient tumors had pathological responses [205]. Final analysis of the NICHE trial showed a 100% pathological response rate. Among 32 dMMR patients, 31 (97%) had a major pathological response and 1 had a partial response. In the pMMR (mismatch repair proficient) cohort, among 30 patients, only 9 (30%) had a pathologic response. No disease recurrence or grade 4 immune-related adverse events were seen in the dMMR cohort [206].

According to results of the NICHE-2 trial, a pathologic response was observed in 99% (106 of 107) of patients, including 95% with a major pathological response, 4% with a partial response, and 67% patients had a complete pathological response. No disease recurrences were observed at the 13-month median follow-up [207]. Results of the NICHE-2 trial presented at the ESMO Congress 2022 showed a difference in pCR between sporadic and Lynch-associated CRC (38 (58%) vs. 25 (78%); *p* = 0.056) [207].

3. Dostarlimab

The efficacy of another anti-PD-1 drug, dostarlimab, in pretreated patients with dMMR solid tumors was tested in the GARNET trial, resulting in an ORR of 36.2% for CRC patients, with 3 complete and 22 partial responses [208,209]. The most impressive results have been obtained with dostarlimab in neoadjuvant settings in patients with dMMR stage II or III rectal cancers. The study design implied the administration of standard chemoradiotherapy and surgery for patients who did not achieve a complete response after neoadjuvant dostarlimab. However, all 12 recruited patients had complete clinical responses, so no surgery was required. During 6 to 25 months of follow-up, no recurrence had been reported [42].

4. Other currently not FDA-approved ICIs for MSI CRC

The efficacy of avelumab in the second-line setting in patients with MSI CRC was evaluated in the SAMCO-PRODIGE trial, demonstrating an ORR of 28% (*n* = 61), as compared to an ORR of 30% in the chemotherapy group (*n* = 61). However, avelumab had better 12- and 18-month PFS rates (19% and 9% in the control group and 31% and 27% in the avelumab group (*p* = 0.025)) [210]. In the AVETUX trial evaluating the efficacy of avelumab and cetuximab in combination with FOLFOX in previously untreated patients with metastatic CRC, only two patients had MSI. One of them achieved a partial response and another one had stable disease [211,212].

The study of durvalumab in pretreated MSI solid tumors showed an ORR of 23% across all patients (*n* = 62) and 22% in patients with CRC (*n* = 36) [213]. Another study, NCT03435107, with a similar design but a different dosage regimen, yielded an ORR of 42% among 33 patients with MSI/dMMR (*n* = 30) or *POLE*-mutated tumors (*n* = 3) [214].

There were no published systematic studies of atezolizumab efficacy in MSI CRC. In the IMblaze 370 study, 6 out of 363 enrolled patients had MSI. Responses were observed in 2 of the 3 patients in the atezolizumab and cobimetinib arm, and in 1 of 3 in the atezolizumab monotherapy group [215]. The MyPathway basket study also showed atezolizumab activity in different types of MSI tumors: among 11 patients with MSI and TMB-H, 6 (54.5%) had a confirmed ORR [216].

Nivolumab in combination with relatlimab is currently being investigated in patients with MSI resistant to prior PD-(L)1 inhibition. Interim analysis of 13 patients showed 1 complete response in a patient with CRC, 1 partial response in a patient with small bowel cancer, and 5 patients achieved stable disease [217].

Tremelimumab in combination with durvalumab and metronomic oral vinorelbine was tested in 30 patients with MSI and/or TMB-H solid tumors. One complete response, nine partial responses, and five stable diseases were observed [218].

Another drug, cemiplimab, has not yet been studied in the context of microsatellite instability.

Therefore, MSI CRC are highly sensitive to immunotherapy with different types of ICIs. Their efficacy tends to increase in earlier lines of therapy. In the neoadjuvant settings, ICIs demonstrate a response rate close to 100%. According to the recent systematic review, clinical trials did not reveal a difference in response between sporadic and Lynch-associated CRC [219]. In two later published studies conducted in neoadjuvant settings and described above, one showed a numerically high, but statistically insignificant, difference in pCR [210], while the other study did not reveal a difference [199]. The absence of a significant difference in responses to ICI between a sporadic and Lynch-associated MSI is consistent with the lack of a response association with the *BRAF* mutation—a surrogate biomarker of sporadic MSI. Although pembrolizumab is approved for first-line treatment of patients with MSI CRC, interim results of the KEYNOTE-177 study indicate that in *KRAS* mutant patients, combined anti-PD1/anti-CTLA-4 therapy is preferred over anti-PD1 monotherapy [197]. Additionally, PD-L1 inhibitors avelumab and durvalumab have been shown to result in a numerically lower ORR: 28% and 22%, respectively, as compared to PD-1 inhibitors.

#### 5.2.2. Tumor Types other Than Colorectal

The second most common cancer with high prevalence of the MSI phenotype is endometrial carcinoma (EC). The efficacy of pembrolizumab for the treatment of metastatic MSI EC was established in the KEYNOTE-158 trial. In the pretreated patients (*n* = 79), the ORR was 48%, median PFS was 13.1 months, and median OS was not reached [220]. Pembrolizumab in combination with lenvatinib showed a numerically lower ORR in the KEYNOTE-775 trial. The study evaluated the efficacy of this combination in patients with pretreated EC with or without dMMR. In the dMMR cohort (*n* = 65), the ORR was 40%, compared to 12% in the chemotherapy cohort (*n* = 65) [221]. In the pMMR cohort (*n* = 346), the ORR was 30% in the experimental group and 15% in the chemotherapy group (*n* = 351) [221].

The efficacy of dostarlimab in dMMR/MSI or MMR-proficient/MSS EC was evaluated in the GARNET study. The ORR was 45.5% in the dMMR/MSI group (*n* = 143) and 15.4% in the MMR-proficient (MMRp)/MSS group. PFS probability at the 12th month was 46.4% and 29.4%, respectively. Median OS was not reached in the dMMR/MSI and was 16.9 months in the MMRp/MSS group [222].

Another tumor type with a high occurrence of MSI is gastric cancer. Post hoc analysis of three clinical trials, KEYNOTE-059, KEYNOTE-061, and KEYNOTE-062, showed greater activity of pembrolizumab in MSI advanced gastric or gastroesophageal junction tumors. More than half of the patients had objective responses, while the ORR in chemotherapy groups was significantly lower (17% in KEYNOTE-061 and 37% in KEYNOTE-062) [223]. The GERCOR NEONIPIGA study showed that nivolumab plus ipilimumab in neoadjuvant settings leads to a 58.6% (17 of 29) pathological complete response rate in patients with resectable gastric/gastroesophageal junction (GEJ) adenocarcinoma [224].

The most complete data for other types of cancers were obtained in the KEYNOTE-158 trial (listed in Table 2). The lowest ORR of 18% was observed in pancreatic cancer patients, while other cancers demonstrated an ORR of more than 30%. It should also be noted that despite an ORR of 33% observed in patients with ovarian cancer, 12 of 24 patients had progressive disease, i.e., a high level of primary resistance to immunotherapy [44].

There are some doubts about the efficacy of immunotherapy in adjuvant settings. From the theoretical point of view, immunotherapy may be more effective in the presence of large amounts of tumor cells in the organism [225]. Thus, this approach appears to be more potent either before primary tumor resection or in the presence of metastatic disease. These considerations were confirmed in the mouse models [226] and in melanoma patients [227,228,229]. According to the Clinical Trials Database, a number of clinical trials of adjuvant immunotherapy in MSI cancers were initiated, and the design of two of them was published [230,231]. One of them—POLEM—in stage 3 MSI or *POLE* mutant CRC patients was recently terminated due to recruitment challenges [232].

In summary, the vast majority of cancer types with features of MSI respond to the immunotherapy. While ICI efficacy in CRC, EC, and gastric cancer is well-established, for other types of cancer, immunotherapy is plausible when other standard options have been exhausted.

## 6. Mechanisms of Resistance to ICI

As previously shown, a significant proportion of MSI patients do not respond to ICI therapy. About 25% of MSI CRC cases have intrinsic resistance to immunotherapy [43]. Several mechanisms have been proposed to explain this observation.

It has been supposed that copresence of mutations in oncogenes might play a role in therapy resistance to ICI. Numerous prospective clinical trials have tested the effect of *BRAF* and *RAS* mutations on the response to ICI therapy in dMMR/MSI CRC patients but all except one described above [197] did not find a significant association [41,196,197,200,201,233,234]. A small retrospective study has linked *BRAF* mutations to worse 1-year and 2-year PFS rates in 60 dMMR CRC patients (40% vs. 73.3%, 26.7% vs. 73.3%; *p* < 0.001) [235]. However, according to a recent meta-analysis, *BRAF* mutations do not influence the response to ICI, while remaining a negative prognostic biomarker [236]. The association between oncogenic mutations with ICI resistance in MSI CRC was not confirmed for *B2M*, *HLA*, and *JAK1/2*, although these genes were found to be frequently mutated in MSI CRC [46,47].

TMB can be considered as an independent biomarker for ICI benefit in MSI tumors. A few retrospective studies showed that TMB correlates with ICI response in MSI gastrointestinal cancers [237,238,239]. Theoretically, MSI tumors should have a large number of mutations, i.e., should be tumor mutational burden-high (TMB-H). In real practice, there is a discordance between these biomarkers as well as discordance between studies. For example, one large study revealed that in 2179 MSI cancer samples, 17.9% were tumor mutational burden-low (TMB-L) (which was defined as <20 mutations/mb) [150]. Another study reported the absence of TMB-L cases (defined as <30 mutations/mb) between 32 CRC samples with MSI, as proven by both IHC and PCR approaches. At the same time, among 56 TMB-L samples, 50 were identified as pMMR/MSS, while 6 samples had discordant MSI status: 5 were determined as dMMR by IHC only, and 1 as MSI by PCR only. IHC retesting resulted in redefining four of five dMMR cases as pMMR [240]. The discordance between these two studies can be explained by different methods of TMB evaluation. In the first study, Foundation One was used, which excludes truncating mutations in tumor suppressor genes [169]. While frameshift mutations are enriched in MSI tumors, this method may underestimate the number of antigen-producing mutations. The second study used a method that takes into account frameshift mutations, explaining the high concordance of its results with the MSI status. This study also revealed a problem of false-positive dMMR recognition, which can contribute to the number of cases resistant to ICI. In addition, it can be assumed that more correct methods for TMB calculation will allow more accurate determination of subgroups of patients resistant to immunotherapy.

Apart from mechanisms involving machinery of the adaptive immune system, MSI tumors may trigger an immune response through accumulation of cytosolic DNA. MLH1 deficiency leads to Exo1 hyperactivity and increased DNA excision. Nuclear DNA release into the cytoplasm activates an innate immune signaling pathway, the cGAS-STING system, which induces expression of type I interferons and other immune molecules. They slow down cell proliferation and promote immune cell infiltration in the affected area. Retrospective analysis showed that patients responding on immunotherapy had higher cGAS/STING expression. Thus, disruption of the cGAS/STING pathway can be considered as one of the mechanisms of resistance to immunotherapy [20,241].

The tumor microenvironment, including tumor-infiltrating lymphocytes (TILs), represents a promising area for finding predictors of the response to immunotherapy. MSI CRC tumors are characterized by significant lymphocyte infiltration [242]. A small study conducted in 30 MSI CRC patients showed an ORR of 70.6% in patients with a high number of TILs, compared to 42.9% in patients with a low number of TILs (odds ratio = 3.20, *p* = 0.0291) [239]. Lymphocyte subsets’ composition is usually evaluated with the Immunoscore assay based on the quantification of CD3+ and CD8+ cells [243]. It was shown that MSI CRC tumors have significantly higher Immunoscores than MSS: 0.57 (SD 0.97) in the general population (*n* = 550) vs. 2.4 in MSI tumors (*n* = 41) (*p* < 0.0001) [244]. Inflammation is often observed in patients with CRC. A recent study showed that this medical complication is associated with an immunosuppressive microenvironment, a higher neutrophil–lymphocyte ratio, and a worse response to ICI [245].

Gut microbiome composition is associated with ICI response in melanoma patients [246,247]. It was shown that MMR-deficient and -proficient CRC tumors differ in gut microbiome composition [248]. However, the role of microbiota in immunotherapy resistance in MSI-H cancers largely remains underexplored [49]. A retrospective study of 57 MSI-H/dMMR CRC patients showed no association between antibiotic exposure and ICI response, indicating a limited role of gut bacteria [249].

More fundamental mechanisms of ICI resistance may be explained in the context of immunoediting theory. According to this theory, tumor cell subclones harboring immunogenic neoantigens are eliminated by the immune system during tumor progression [250]. This might explain the difference between the prevalence of MSI in early and advanced stages [84] as well as the difference in response between neoadjuvant and palliative therapy described above.

Thus, to date, precise mechanisms of MSI tumors’ resistance to immunotherapy remain unclear and there are no known predictive biomarkers ready for implementation in the clinic. The fact that virtually all tested non-metastatic MSI tumors respond to ICI in neoadjuvant settings suggests that intrinsic resistance may be acquired during tumor evolution to the metastatic state.

## 7. Conclusions and Future Perspectives

MSI occurs in many types of tumors, both those associated with Lynch syndrome (colorectal cancer, endometrial cancer, cancer of the small intestine, and gastric cancer) and sporadic, but with a lower incidence. MSI is a key diagnostic biomarker for Lynch syndrome and is one of the best predictive biomarkers of immune checkpoint inhibitors’ (ICIs) efficacy. Since the FDA approved the first drug in the class of ICI, the treatment landscape of many cancer types has significantly improved. Recognition of MSI as a tissue/site-agnostic biomarker for pembrolizumab became an important milestone in the development of precision medicine. Among other types, colorectal and endometrial cancers are the most well-studied in terms of the predictive role of MSI in relation to the efficacy of ICI.

However, despite that ICIs typically result in high objective response rates, about a quarter of MSI metastatic cases demonstrate primary resistance, i.e., progress after the start of treatment. The underlying causes for this resistance have been extensively studied. Mutations in certain signaling pathway genes, correlation with TMB, the tumor microenvironment, gut microbiome, and tumor immunoediting have been suggested as possible causes, but to date, precise mechanisms of resistance remain elusive. Thus, further studies are needed, especially to confirm the efficacy of immunotherapy for the MSI rarer, less studied tumors, as well as to reveal the intrinsic difference between individual patients responsive or resistant to treatment.

## Figures and Tables

**Table 1:** dMMR/MSI-related indications for immune checkpoint inhibitors.

Checkpoint	Drug	Indications for MSI and/or dMMR Tumors
CTLA4	Ipilimumab (in combination with nivolumab)	MSI/dMMR colorectal cancer
	Tremelimumab	Indications do not include MSI/dMMR tumors
PD1	Nivolumab	MSI/dMMR colorectal cancer
	Pembrolizumab	All types of MSI/dMMR tumors (site-agnostic) MSI/dMMR colorectal cancer MSI/dMMR endometrial cancer
	Cemiplimab	Indications do not include MSI/dMMR tumors
	Dostarlimab	All types of dMMR tumors (site agnostic) dMMR endometrial cancer
PD-L1	Atezolizumab	Indications do not include MSI/dMMR tumors
	Avelumab	Indications do not include MSI/dMMR tumors
	Durvalumab	Indications do not include MSI/dMMR tumors
LAG3	Relatlimab	Indications do not include MSI/dMMR tumors

**Table 2:** Response rates of MSI/dMMR tumors to ICI therapy in selected clinical trials.

Study	Total Number of Patients	Drug	ORR or pCR (for Neoadjuvant Settings)	Lynch Syndrome Accounted	*BRAF/RAS* Status Evaluated	PD-L1 Expression Status Evaluated by IHC
Tissue/Site-Agnostic Treatment in Pretreated Metastatic Patients						
KEYNOTE-016, KEYNOTE-164, KEYNOTE-012, KEYNOTE-028, KEYNOTE-158 [39]	149 patients: 89 CRC 14 EC 45 other	Pembrolizumab	40%	No	Yes	Yes
KEYNOTE-158 [40]	233 non-CRC	Pembrolizumab	34%	No	No	Yes
GARNET [193] (including POLE mutant)	209 non-EC	Dostarlimab	43%	No	No	Yes
Colorectal cancer						
Late lines						
KEYNOTE-016 [105]	40	Pembrolizumab	52%	Yes	No	Yes
CheckMate-142 [200]	119	Nivolumab plus Ipilimumab	55%	Yes	Yes	Yes
NCT01693562 [213]	36	Durvalumab	22%	No information	No information	No information
NCT03435107 [214]	33	Durvalumab	42%	No	Yes	No
SAMCO-PRODIGE [210]	61	Avelumab	28%	No	Yes	Yes
First line						
KEYNOTE-177 [196]	153	Pembrolizumab	45%	No	Yes	No
CheckMate-142 [41]	45	Nivolumab plus Ipilimumab	69%	No	Yes	Yes
Neoadjuvant settings						
NICHE [205]	20	Nivolumab plus Ipilimumab	60%	Yes	Yes	Yes
NICHE-2 [207]	107	Nivolumab plus Ipilimumab	67%	No information	No information	No information
NCT04165772 [42]	12	Nivolumab plus Ipilimumab	100%	Yes	Yes	Yes
Endometrial cancer						
KEYNOTE-158 [220]	79	Pembrolizumab	48%	No	No	Yes
GARNET [153,222]	143	Dostarlimab	46%	No	No	Yes
Gastric cancer						
KEYNOTE-059, KEYNOTE-061, KEYNOTE-062 [223]	7 27 50	Pembrolizumab	57% 47% 57%	No	No	Yes
KEYNOTE-158 [44]	42	Pembrolizumab	31%	No	No	Yes
Small intestine cancer						
KEYNOTE-158 [44]	25	Pembrolizumab	48%	No	No	Yes
Ovarian cancer						
KEYNOTE-158 [44]	24	Pembrolizumab	33%	No	No	Yes
Cholangiocarcinoma/biliary tract cancer						
KEYNOTE-158 [44]	22	Pembrolizumab	41%	No	No	Yes
Pancreatic cancer						
KEYNOTE-158 [44]	22	Pembrolizumab	18%	No	No	Yes
